# Effect of Using Faba Bean and/or Italian Ryegrass in Total Mixed Rations to Dairy Cows

**DOI:** 10.3390/ani13010108

**Published:** 2022-12-27

**Authors:** Silvia Baizán, Adela Martínez-Fernández, Fernando Vicente

**Affiliations:** Servicio Regional de Investigación y Desarrollo Agroalimentario (SERIDA), Carretera AS-267, PK. 19, 33300 Villaviciosa, Asturias, Spain

**Keywords:** legume, silage, milk, fatty acids, sustainable livestock

## Abstract

**Simple Summary:**

The dairy sector faces toward the challenge of increasing its production due to the growing demand for food. At the same time, it should be more efficient in the use of natural resources, replacing usual agricultural practices for others that are more sustainable. Feeding systems based on forages are economically more competitive than those based on concentrates. Therefore, to improve the dairy sector profitability, complying at the same time with environmental requirements, the change actions should be oriented to modify the traditional Italian ryegrass crop by the inclusion of other species with similar yield, but more sustainably and in a way that can be integrated into the dairy cows’ feed without affecting milk yield and quality. Based on the above, this study was proposed in order to identify a winter crop to supply a quality livestock feed, which involves a reduction of production inputs, allowing the recycling of nutrients and the preservation of natural resources. It is intended that the results obtained will help farmers to achieve competitiveness in the dairy sector linked to quality, food safety and innovation, reducing production costs, increasing profitability, and improving the functional components of milk, seeking economic and environmental sustainability of the dairy production sector.

**Abstract:**

The livestock sector must find solutions to reduce the feeding costs and meet the challenge for a more sustainable production system in line with the European Green Deal requirements. The aim of this study was to evaluate the effect of including legume silage on voluntary intake in dairy cows, milk production, and composition. Three total mixed rations (TMR) based on faba bean (FB), Italian ryegrass (IR), or faba bean–Italian ryegrass intercrop (FBIR, 60:40) silages were used in feeding trials of dairy cows during two consecutive years. Nine Friesian cows were randomly allocated in three groups, following a 3 × 3 Latin square design with three diets for three periods. TMR were offered ad libitum in addition to eighteen hours of grazing daily and extra concentrate during milking. No differences were observed in dry matter intake. Diets did not affect milk production or composition, except for urea content, with a higher urea excretion in FB diet. Fatty acid profile was different in milk from cows feeding FB, with a significantly lower content of saturated fatty acids and a higher content of conjugated linoleic acid than milk produced with FBIR and IR diets.

## 1. Introduction

Grass grazed is the most cost-effective way of feeding dairy cows [1]. In addition, diets based on pasture and grass silage can improve milk nutritional quality, shifting their fatty acid (FA) composition toward less saturated fatty acids (SFA) and more polyunsaturated fatty acids (PUFA), especially omega-3 groups [2]. Forage legumes show a higher transfer efficiency of PUFA to bovine milk fat in comparison with grasses [3]. Consequently, the inclusion of legume silage in dairy cow rations would allow improvement in the milk lipid profile from the point of view of human health. This fact, together with the need to reduce costs, particularly for dietary protein and soil fertilizers, means that legume silages represent an important and interesting tool for dairy farmers [4]. The Italian ryegrass is a winter crop commonly used in many European dairy farms [5]. However, because the Italian ryegrass has high fertilization requirements, it could be replaced by other winter crops such as some legumes, which have lower nitrogen requirements. At this point both, faba bean in monoculture and in intercrop with Italian ryegrass are alternative and competitive crops to Italian ryegrass in monoculture [6]. The yields obtained with these alternatives are comparable to Italian ryegrass but with the advantage that they need less mechanization as a result of a single cut harvest system, besides presenting good ensilability characteristics and multiple ecosystem services [7]. However, before recommending these crops to farmers, it is necessary to verify the effect they may have on intake, and how they influence the production and composition of milk.

Based on previous works [6,7], this study was raised with the intention that the results obtained can help farmers to achieve the competitiveness of the milk-producing sector linked to quality, food safety and innovation, reducing production costs, increasing profitability, and improving the functional components of milk, seeking economic and environmental sustainability of the dairy-producing sector under the conditions of the Common Agricultural Policy (CAP). The aim of this study was to evaluate the effect of the inclusion of faba bean legume silage in rations for dairy cows on the voluntary intake and on the production and composition of the milk in comparison with the Italian ryegrass silage commonly used.

## 2. Materials and Methods

### 2.1. Animals and Diets

Two trials were conducted in the SERIDA experimental farm located at Villaviciosa, Asturias, Spain (43°28’20’’ N, 5°25’10’’ W and 10 m above sea level) in two consecutive years. Nine Friesian dairy cows were used in each trial. Animals had 648 ± 59 kg bodyweight, 2.7 ± 1.58 lactations, 42 ± 5 days in milk, and 29.6 ± 2.00 kg milk day^−1^ at the first trial, and 664 ± 62 kg bodyweight, 2.5 ± 1.93 lactations, 30 ± 7 days in milk, and 33.6 ± 5.00 kg milk day^−1^ at the second one. The trials were made in accordance with current Spanish and European legislation (RD 53/2013, EU directive 2010/63/UE) for protection of animals used for scientific purposes. In each trial, cows were allocated into three groups of three cows each and were fed ad libitum with different TMR formulated according to NRC [8]: (1) TMR based on Italian ryegrass silage (IR); (2) TMR based on faba bean silage (FB) and (3) TMR based on faba bean–Italian ryegrass intercrop silage (60:40) (FBIR). The conditions for growing, harvesting, and ensiling of these forages are described in a previous paper [7]. The silages were sampled before starting the experiments to formulate the TMR according to their nutritive value. The TMR were made using the Italian ryegrass silage or faba bean silage or faba bean–Italian ryegrass intercrop silage, cereal straw (Dry matter (DM): 912.8 g kg^−1^; Organic matter (OM) 934.1 g kg^−1^ DM; Crude protein (CP): 31.4 g kg^−1^ DM; Neutral detergent fiber (NDF): 767.4 g kg^−1^ DM; Acid detergent fiber (ADF): 456.9 g kg^−1^ DM, and Metabolizable energy (ME): 6.81 MJ kg^−1^ DM), and a compound feed (DM: 886.1 g kg^−1^; OM: 933.4 g kg^−1^ DM; CP: 189.9 g kg^−1^ DM; NDF: 225.8 g kg^−1^ DM; ADF: 97.5 g kg^−1^ DM, and ME: 12.71 MJ kg^−1^ DM). The ingredient compositions of TMR and compound feed are detailed in Table 1. TMR were supplemented daily with grazing for 18 hours in polyphyte meadows. All cows grazed together in a rotational grazing system overnight and for 6 daylight hours in three 1.5 ha paddocks to maintain a stocking rate of 2.5 cows/ha. An extra concentrate (EC) was supplied during the milking sessions as an energetic supplement adjusted to the production level of each cow in both trials (1 kg of fresh matter daily as a base dose that was increased at a rate of 0.2 kg per kg of milk produced above the average milk yield of the herd, up to a maximum of 6 kg of extra concentrate). Cows always had available fresh water and mineral blocks composed of NaCl (970 g kg^−1^), MnO_2_ (70 mg kg^−1^), CuSo_2_·H_2_O (50 mg kg^−1^), ZnO (40 mg kg^−1^), FeSO_4_·H_2_O (40 mg kg^−1^), KI (40 mg kg^−1^), C_4_H_6_CoO_4_ (30 mg kg^−1^), Na_2_SeO_3_ (2 mg kg^−1^), and inert excipient.

### 2.2. Experimental Procedure

In both trials, cows were assigned following a 3 × 3 Latin square design with three diets offered to three groups of three cows (randomly distributed) in three consecutive feeding periods. Each trial consisted of three periods of 21 days each, including 14 days for diet adaptation and 7 days for data collection. Animals were weighed at the first and the last day of each data collection period. TMR were elaborated daily, and the intake of the individual cows was automatically recorded daily by an electronic weighing system integrated to scale pen by a computerized system. The intake of extra concentrate was recorded daily by the automatic feeder integrated in the milking system. Grass intake was estimated with the animal performance method [9]. Briefly, energy requirements were recorded as net energy (NE, Mcal day^−1^) requirements for maintenance, lactation, bodyweight changes, walking, and grazing. The NE from pasture intake was estimated as total NE requirements minus the NE supplied by the TMR and extra concentrate intakes. All TMR were sampled daily during the data collection period. Extra concentrate was sampled at the beginning of each data collection period. The grazing meadows were sampled the previous day of the data collection period by tracing a diagonal transect across the available grazing area to measure the herbage availability. The grass sample consisted of a sampling area of 1 m^2^ per hectare composed by five subsamples of the forage cut at 6 cm of height over a surface of 2 × 0.1 m with a hand mower. Cows were milked twice daily from 07:00–08:00 h and 19:00–20:00 h. After morning milking, the cows remained indoors until 12:30 h and then moved to their corresponding plot until evening milking. After evening milking, the cows were moved to their corresponding plot, where they stayed until next morning milking. Milk production by cow was recorded every day during the data collection period. Samples of milk were taken during both milking sessions of the data collection period; samples were added with azidiol for macronutrients analyses and without azidiol for FA profile. Energy-corrected milk (ECM, with 4.0% fat and 3.4% protein) for every cow was calculated based on the equation [10]:

ECM (kg) = milk (kg) × (0.38 × fat% + 0.21 × protein% + 1.05)/3.28(1)

### 2.3. Analytical Procedures

Silages, grass, and TMR samples were dried at 60 °C for 24 h and ground at 0.75 mm. The extra concentrate was ground at 1 mm. All samples were analysed by NIRS (Foss NIRSystem 5000, FOSS, Silver Spring, MD, USA) for DM, OM, CP, NDF, and ADF. In addition, in extra concentrate and TMR, the ether extract (EE), the starch content (STR), and the net energy of lactation (NEI) were determined. The energy content was estimated in all samples according to NRC [8]. The FA content of silages and TMR was analysed according to Palmquist and Jenkins [11], using a Varian 3800 GC4000 mass spectrometer (Varian Inc., Palo Alto, CA, USA) with a CP-Sil 88 column (100 m × 0.25 mm, 0.20 µm internal diameter; Varian Inc.). Peaks were identified by comparison of retention times and mass spectra obtained between samples and the methyl ester standards: GLC-463 (52 FA methyl esters mix) and UC-61-M (t10c12 18:2) of Nu-Check (Nu-Check-Prep Inc., Elysian, MN, USA) and methyl 9(Z), 11(E)-octadecadienoate of Matreya (Matreya LLC, Pleasant Gap, PA, USA). Methyl nonadecanoate was used as internal standard (Sigma-Aldrich Inc., St. Louis, MO, USA). Milk samples were analysed for fat, protein, lactose, solids non-fat, and urea using MilkoScan FT 6000 (FOSS Tecator, Hillerød, Denmark). Milk fat was extracted according [12], and the Christie methodology [13] with modifications [14] for FA analysis was used.

### 2.4. Statistical Analysis

Statistical analysis was performed using the R statistical package [15]. Variances homogeneity was checked a prori using Levene’s test. FA results of TMR were contrasted by an analysis of variance (ANOVA) using a simple linear regression model in which diet was considered as the main factor and year as a random factor. Post hoc comparisons of means between treatments were performed by applying Duncan’s test at a significance level of *p* < 0.05. Results of live weight, dry matter intake and production, macronutrient composition and milk FA profile were contrasted by ANOVA using a mixed linear regression model considering diet and period as fixed effects and cow and year as random effects. Post hoc comparisons of means between treatments were performed using a Bonferroni test, at a significance level of *p* < 0.05. For heteroscedastic data, the Kruskal–Wallis test was performed, and post hoc comparisons of means between treatments were also carried out, also applying a Bonferroni test with the same level of significance as in the previous case (*p* < 0.05).

## 3. Results

The characteristics of the silages used are detailed in Table 2. The faba bean silage has higher dry matter content than Italian ryegrass silage alone or in intercrop with faba bean. The silages with legumes have higher concentration of both neutral and acid detergent fibers than Italian ryegrass silage. Consequently, the organic matter digestibility and energy content were lower. Grass height, pasture availability, and meadow botanical composition for grazing in both feeding trials are shown in Table 3. The botanical composition presented a variable distribution of grass species between years and periods. It was not possible to complete the botanical composition of the third period of the second trial due to a problem with the herbage samples’ conservation, so these results were not included. The main grass species observed were *Lolium perenne* L., *Agrostis* spp., *Bromus* spp., *Paspalum* spp., and *Dactylis glomerata* L. The most abundant legume was *Trifolium repens* L., and species of *Taraxacum* genus were the most numerous within dicotyledons. Grass height remained within recommended values for rotational grazing (between 15 and 20 cm) in all periods of two trials. Grass availability remained above the minimum suitable for grazing (of the order of 1100 kg DM ha^−1^) except for the last two periods of the second trial (P2 and P3 of the year 2016) in which it was around 900 kg DM ha^−1^.

Table 4 shows the nutritive value of TMR, concentrate, and grass in each trial. DM and NDF contents were higher in rations with FB silage in both years of the study. CP content was similar for the three rations tested in both years. Differences were observed in EE of rations with FB and FBIR in the first year of study compared to the second. The energy content was the same for three rations in the first year, while in the second year the ration with IR silage showed a slightly higher content compared to the other two rations.

The main FA present in TMR were palmitic, oleic, linoleic, and linolenic acids, constituting about 93.87, 91.84, and 91.51% in TMR made with IR, FB, and FBIR silage, respectively (Table 5). Linolenic acid was predominant FA in TMR made from IR silage, while in rations formulated with FB and FBIR silage, the most abundant was linoleic acid. There was a trend to be significant the differences between rations with IR and FB in the concentrations of palmitic acid (*p* = 0.086) and linoleic acid (*p* = 0.077). No significant differences were observed between TMR in oleic. Diet with IR presented a higher content of linolenic acid (*p* < 0.05) compared to the other two rations.

Table 6 shows initial and final cows’ body weights, dry matter intakes, and effects of TMR tested on milk production and composition. No significant differences were observed in live weight, neither at the beginning nor at the end of the trials, nor in the daily weight gain of animals depending on diet. Similar dry matter intakes of TMR, concentrate, and grass were observed (*p* > 0.05). Milk yield was lower in cows feeding FB and FBIR than feeding IR treatment. In milk macro-components, significant differences (*p* < 0.001) were only observed in urea content. Milk produced from TMR with FB silage had the highest concentration, and IR silage the lowest, while FBIR treatment had intermediate urea values. Table 7 shows milk FA profile obtained as a function of each diet and the Table 8 the sum and ratios of FA according to the number of double bonds. The main FA observed were palmitic, myristic, oleic, and stearic acids, with approximately 77.47, 76.95, and 78.03% for TMR with IR, FB, and FBIR, respectively. Palmitic acid content was higher (*p* < 0.05) in milk produced with IR and FBIR silage rations than FB silage rations. Myristic acid concentration was similar for all diets and oleic and stearic contents were higher (*p* < 0.001) in milk of FB silage rations. Total polyunsaturated fatty acids (PUFA) were higher (*p* < 0.001) in milk FB silage rations. Omega-3 (ω-3), omega-6 (ω-6), FA, and the ω-3/ω-6 ratio did not present significant differences among diets since 98% of the ω-3 corresponded to the linolenic acid and 98% of the ω-6 to linoleic acid, which were similar between treatments.

## 4. Discussion

In this study, meadows botanical composition was 77.26% of grasses, 3.91% of legumes, and 5.87% of other families. The 12.40% remain was attributed to unidentifiable plant remains and dead matter. Meadows have a balanced botanical composition when the grasses percentage is between 50 and 70% and legumes and other families percentage is between 10 and 30% [16]. Therefore, the legumes population was below the optimal value recommended for this botanical family. These results agree with other studies in adjoining meadows [17], in which legumes represented entirely by *Trifolium repens* L. constituted 1.33% of the total, while grasses identified constituted 89.13%. This low contribution of legumes to grassland biomass is due to the fact that clover is a very sensitive species to compete for light and nutrients [18]. Clover establishment and maintenance to achieve an adequate balance in grasslands is difficult in regions of the Atlantic arc such as northern Spain, characterized by temperate and humid climates and in which it is usual for the production *Trifolium repens* L. in association with *Lolium perenne* L. to not exceed 25–30% of the total production of grassland, which is between 10 and 13% of the total dry matter [19]. The differences observed between years in grass production are due to soil and weather conditions of the area of the study, as has been observed in previous agronomic studies [6,7].

Nutritional and energetic grass value was highly variable between periods and trials, probably due to phytological difference between meadows. Some studies pointed out that floristic diversity influences grassland nutritional value due to differences in the chemical composition and digestibility of the individual species and the different phenological states [20,21]. In addition, there are numerous factors influencing grassland botanical composition and, therefore, nutritional grass value [22], such as soil type, water availability, variation annual in climatic conditions [23], nutritional status, and management practices [24,25]. In other studies carried out in these meadows, the variations found in grass chemical composition were attributed to a logical evolution of plant maturity and/or to the use of different meadows in each trial period [26] or due to the differences observed to percentage contribution of dominant species [17].

Green forage FA profile from the same meadows used in this study has been well-defined previously [2,17,26,27]. Due to the FA homogeneity found in these previous studies, the FA profile was not analyzed again. The main FA of these forages are palmitic, oleic, linoleic, and linolenic acids. All of them present proportions around 20, 2, 17, and 58 g 100 g^−1^ of FA for each of them, respectively. Additionally, the concentrate was homogeneous in terms of nutritional value in both trials (2015 and 2016) as expected in a commercial product. According to previous studies, the concentrate had a high content of palmitic acid, around 44 g 100 g^−1^ of FA, followed by 29 and 18 g 100 g^−1^ of FA of oleic and linoleic acid, respectively, and a low concentration of linoleic acid, about 0.3 g 100 g^−1^ FA [2,17,26,27].

TMR rations supplied in both trials were formulated to be isonitrogenous and isoenergetic according to results of crude protein and metabolizable energy of silages and the other ingredients. In the first feeding trial (year 2015), the protein and energy contents were similar in the three rations tested. However, in second trial (year 2016), the higher amount of concentrate used in ration made with IR silage to reach the same level of crude protein as in the other rations resulted in a slightly higher energy value (1.50 vs. 1.39 vs. 1.39 Mcal kg^−1^ DM for the IR, FB, and FBIR rations, respectively). Similar results were observed in rations made with Italian ryegrass silage compared to rations made with faba bean and rapeseed intercrop silage [17]. These incidents in rations formulation were due to the fact that faba bean forage has more protein than Italian ryegrass, but less energetic value, so it was necessary to increase the amount of faba bean forage to formulate balanced rations. The faba bean can reach a height of 1.5 to 2 m and whose stems have more than 50% of acid detergent fiber and more than 10% lignin. This is reflected in the lower percentage of organic matter digestibility found in FB and FBIR silages compared to IR silage.

FA content in TMR should be proportional to FA content of silages used because they are the main ingredient, but slight differences were observed, probably derived from the rations elaboration process. The most notable differences were observed in oleic and linolenic acid content. While oleic acid content was increased in TMR compared with silage (1.54; 4.35 and 2.50 g 100 g^−1^ of FA for the IR, FB, and FBIR silages, respectively, vs. 12.44; 16.42 and 15.65 g 100 g^−1^ of FA for the rations with IR, FB, and FBIR, respectively), linolenic acid showed a significant decrease in its concentration compared with silage used for the preparation of ration (60.43; 24.06 and 37.99 g 100 g^−1^ of FA for the IR, FB, and FBIR silages, respectively, vs. 27.85; 8.23 and 11.80 g 100 g^−1^ of FA for IR, FB, and FBIR rations, respectively). After opening the silo, air and light exposure creates an oxidation process that can cause losses of unsaturated FA content [3]. In a study with corn and grass silage, significant decreases were observed in linolenic, linoleic, oleic acids, and in total FA contents after 24 h exposure [28]. In addition, it must be considered that ingredients of TMR used were mixed by mixer wagon. If this procedure is not carried out with care, it can lead to heating of the ingredients used and thermolabile plant compounds, such as phenolic compounds and FA, can be altered. In the case of FA, the most unsaturated acids are the first to undergo changes, so linolenic acid could have lost its unsaturation and acquired the form of oleic acid, thus increasing the content of one to the detriment of the other.

Legumes, such as faba bean, have bioactive compounds (total phenols and condensed tannins) related to decrease the voluntary intake in some studies. Thus, rations made with an intercrop of faba bean and rapeseed showed a significant reduction in the voluntary intake compared to rations made with Italian ryegrass [17]. However, in our study, no significant differences were observed in the voluntary intake in TMR including legume silages compared to TMR with Italian ryegrass silage. This could be because the tannins’ effects depend on concentration, and this in turn depends on the species, variety, and organ of the plant [29]. In addition, the concentration of condensed tannins after the ensiling process of FB and FBIR decreases significantly [29]. Therefore, the depressive effect of faba bean–rapeseed rations intake described [17] could be because of glucosinolates associated to the rapeseed [30] and not be related to polyphenol content present in faba bean forage since, in our case, there has been no such depressing effect.

Milk production in cows’ feeding rations made with faba bean silage (FB and FBIR) was lower than the production obtained with rations based on ryegrass silage. No differences were observed in milk composition except urea content, in which milk produced with legume silage rations (FB and FBIR) presented a higher content than milk produced with IR silage rations. This fact could be the cause of the lower milk production with these diets since additional energy is required to excrete urea by milk [31]. Although milk urea content was higher in FB diet, the values were within optimal data range (210 and 320 mg urea L^−1^) [32]. Milk urea content reflects intake dietary protein and ruminal metabolism, as well as liver and kidney function, so the elevated levels of this parameter can be attributed to an excess of protein in the diet or to an inefficient use of it [33]. Factors such as protein/energy ratio of the diet or non-degradable/degradable protein ratio in rumen can affect milk urea content [34]. Initially, an increase in this parameter may indicate an unbalanced ration [35] but, in similar studies [17,36] where legume silages (faba bean and pea) were used, higher urea concentrations were also obtained than those observed in milk produced with Italian ryegrass silage, so a high level of urea in milk can also be attributed to the use of silage with high ammoniacal nitrogen content.

In the three diets used, the average percentage of milk protein presented values below the 31 g kg^−1^ recommended for payment for quality. These results are usually related to low energy inputs. The energy intake (as opposed to protein intake) is the main driver of milk protein synthesis [37]. Cows that are in a large negative energy balance (for example, high-producing grass-fed cows supplemented with low levels of concentrate) have low concentrations of milk protein, between 28 and 29 g kg^−1^. The animals used in these trials were in the first and second thirds of lactation when the requirements are higher. The rations were formulated to cover the energy requirements, maximizing forage proportion, and minimizing the concentrate. The ruminal degradation of the concentrates increases propionic acid proportion therefore concentration in the rumen would reduce with the diets used. There is a positive correlation between propionic acid concentration and milk protein [38] because it the availability of amino acids is favoured. If an amino acid is not present in sufficient quantity, protein synthesis is limited [39]. Lactose, solids non-fat, and cell concentrations remained within the optimal data range without differences among treatments.

Milk fat and FA concentrations depends mainly on the diet supplied to cows [3] and the microbial activity in the rumen. It has been shown that polyunsaturated acids intake presents on the grass results into higher oleic, vaccenic, and rumenic acids concentrations in milk fat [40,41]. Since all the cows in in this study intake the same type of grass, the differences identified in lipid profile can be attributed to the type of forage supplied in TMR. Milk from cows feeding FB silage-based rations had a lower total SFA content than milk produced with the other rations (IR and FBIR). This difference was mainly due to a lower concentration of palmitic acid, which has been related to increased blood cholesterol in humans [42]. A lower content was also observed in other FA related to cardiovascular risk such as lauric and myristic acids, although not significantly in the latter. In this way, there is a reduction in short- and medium-chain fatty acids in favour of healthful FA, such as oleic acid. This FA is a known antiatherogenic agent and promotes positive effects on human health [43]. This fact agrees with other studies that obtained an increasing proportion of monounsaturated fatty acids (MUFA) and polyunsaturated fatty acids (PUFA) in milk at the expense of capric, lauric, myristic, and palmitic acids when cows were fed legume silage (red clover) against a mixture of timothy and fescue silage [44] or ryegrass silage [45]. Milk produced with rations based on FB silage presented higher levels of PUFA than milk derived from the other diets, which are considered healthful for human due to their anticancer properties [46], especially linoleic and rumenic acid. In this study, there were no significant differences found between treatments for linolenic acid, probably due to the biohydrogenation of this FA in the rumen. However, milk from cows fed with red clover silage showed an increase in the content of omega-3 (linolenic acid) with respect to that of those fed with grass silage [4]. Despite this fact, milk produced with FB silage-based rations showed greater efficiency transfer of this compound to bovine milk fat compared to rations based on IR silage, since IR silage contained 60.43 g 100 g^−1^ FA vs. 37.99 and 24.06 for FBIR and FB, respectively, and the results in milk were 0.98 g 100 g^−1^ FA vs. 1.04 and 1.08 for FBIR and FB, respectively. These results according to with previous studies showing that legumes present a greater efficiency in the transfer of PUFA from the diet to milk fat compared to grasses [4,44]. These comparisons were made with different clover species (*Trifolium pratense* L., *Trifoliun repens* L.) and with vetch (*Vicia sativa* L.). In a study, it has been observed that when Italian ryegrass silage is replaced by legume silage, especially with faba bean silage, milk fat has a higher proportion of unsaturated fatty acids [36]. In addition, when the grazing is included, the effect in milk composition is greater, improving its quality by reducing the proportion of SFA and increasing unsaturated, monounsaturated, polyunsaturated acids, and conjugated linoleic acid content, thus providing a healthier FA profile. It is now known that essential FA, both from the ω-6 series (especially linoleic and arachidonic acids) and the ω-3 series (linolenic, eicosapentaenoic, and docosahexaenoic acids) are essential for the development and growth and play a key role in disease prevention and management coronary arteries, hypertension, diabetes, arthritis, cancer, and other inflammatory conditions and autoimmune [47]. The ω-6/ω-3 ratio in cow’s milk describes essentially linoleic acid concentrations vs. linolenic acid since they represent the ω-6 and ω-3 more abundantly. Therefore, a lower ω-6/ω-3 ratio is indicative of a forage-based diet [1] and a healthier lipid profile. The expert recommendations indicate that a two-part ratio should be consumed of ω-6 for every ω-3 (2:1). In this study, the lowest relationship corresponded to milk produced with FB silage, although without significant differences compared to the other diets, as a result of the absence of significant differences in linoleic acid since almost all the ω-3 present in milk fat corresponded to linolenic acid, and the same in the case of ω-6. These results agree with results observed in milk from cows feeding with silage of faba bean–rapeseed intercrop than feeding with Italian ryegrass silage where, although there were no significant differences were observed on the individual concentrations in these FA, the atherogenicity and thrombogenicity indices showed a healthier profile in milk [17].

## 5. Conclusions

The inclusion of faba bean silage alone or intercropped with Italian ryegrass (FB and FBIR) in TMR rations did not negatively affect the voluntary intake nor the production and chemical composition of milk compared to the conventional ration made with IR silage alone. Rations for dairy cows including FB silage modified milk FA profile. This legume led to a reduction in the palmitic acid content of milk, increasing MUFA and PUFA contents and showing a healthier FA profile for consumers than milk from cows fed FBIR and IR silages.

## Figures and Tables

**Table 1:** Ingredient composition (g kg^−1^ DM) of the total mixed rations (TMR) used in feeding trials.

	Trial 1 (Year 2015)			Trial 2 (Year 2016)		
	IR	FB	FBIR	IR	FB	FBIR
IR silage	570.1	- ^1^	-	459.1	-	-
FB silage	-	659.5	-		604.6	-
FBIR silage	-	-	632.5	-	-	523.7
Cereal straw	98.7	70.4	56.7	56.6	41.4	47.1
Compound feed ^2^	331.2	270.1	310.8	484.3	354.0	429.2

IR: Italian ryegrass monoculture; FB: faba bean monoculture; FBIR: faba bean–Italian ryegrass intercrop (60:40); DM: dry matter. ^1^ Not included. ^2^ Ingredient composition (g kg^−1^ DM): Maize flakes (440.0); soybean meal (340.0); maize grain (47.3); beet pulp (62.1); palm fatty acid salts (26.3); barley grain (22.0); cotton seed (22.0); sodium bicarbonate (18.5); calcium carbonate (7.0); sodium chloride (6.6); dicalcium phosphate (6.2); mineral-vitamin premix (2.0).

**Table 2:** Nutritional value, metabolizable energy, fermentative parameters, and fatty acid profile of silages used to formulate the total mixed rations.

	IR	FB	FBIR	rsd	*p* (M)	*p* (Y)	*p* (M × Y)
Nutritional value and energy contribution							
Dry matter (g kg^−1^)	165.4 ^b^	289.4 ^a^	180.4 ^b^	12.62	<0.001	0.002	0.736
Organic matter (g kg^−1^ DM)	881.1	866.0	877.4	21.68	0.477	0.010	0.772
Crude protein (g kg^−1^ DM)	130.8	139.5	126.4	8.78	0.065	<0.001	0.011
Neutral Detergent Fiber (g kg^−1^ DM)	493.0 ^b^	615.6 ^a^	617.0 ^a^	28.33	<0.001	0.289	0.258
Acid Detergent Fiber (g kg^−1^ DM)	312.0 ^b^	437.9 ^a^	420.1 ^a^	27.79	<0.001	0.053	0.026
OM digestibility (%)	83.28 ^a^	59.25 ^b^	61.64 ^b^	2.616	<0.001	0.201	0.160
Metabolizable energy (MJ kg^−1^ DM)	11.72 ^a^	8.20 ^b^	8.64 ^b^	0.447	<0.001	0.025	0.258
Fermentation parameters							
pH	4.10 ^b^	4.43 ^ab^	4.58 ^a^	0.266	0.023	0.445	0.005
Ammoniacal N (g kg^−1^ Total N)	89.9	124.1	182.0	61.16	0.064	0.787	0.178
Lactic acid (g kg^−1^ DM)	62.8 ^a^	30.3 ^b^	26.7 ^b^	18.76	0.011	0.933	0.166
Acetic acid (g kg^−1^ DM)	54.8 ^a^	21.4 ^b^	46.6 ^a^	14.30	0.004	0.026	0.049
Propionic Acid (g kg^−1^ DM)	2.3	1.7	02.5	0.155	0.627	0.052	0.523
Butyric acid (g kg^−1^ DM)	10.5 ^b^	8.7 ^b^	32.3 ^a^	15.68	0.041	0.442	0.476
Fatty acids (FA) (g 100 g^−1^ FA)							
Caproic acid	0.52	2.13	2.35	1.553	0.154	0.425	0.108
Heptanoic acid	0.01	0.02	0.04	0.043	0.526	0.135	0.526
Caprylic acid	0.07 ^b^	0.19 ^a^	0.16 ^a^	0.055	0.009	0.001	0.160
Capric acid	0.02 ^b^	0.07 ^a^	0.06 ^a^	0.011	<0.001	0.004	0.145
Lauric acid	4.95	2.63	5.79	2.770	0.209	<0.001	0.231
Myristic acid	0.55 ^b^	0.87 ^a^	0.83 ^a^	0.084	<0.001	0.264	0.011
Pentadecanoic acid	0.11 ^c^	0.32 ^a^	0.22 ^b^	0.023	<0.001	0.537	0.241
Palmitic acid	17.37 ^b^	24.27 ^a^	23.15 ^a^	1.453	<0.001	0.093	0.001
Palmitoleic acid	0.17 ^c^	0.54 ^a^	0.37 ^b^	0.078	<0.001	<0.001	<0.001
Heptadecanoic acid	0.10	0.37	0.22	0.014	<0.001	<0.001	<0.001
Stearic acid	0.92 ^c^	3.00 ^a^	1.94 ^b^	0.086	<0.001	<0.001	<0.001
Oleic acid	1.54 ^c^	4.35 ^a^	2.50 ^b^	0.506	<0.001	<0.001	<0.001
Vaccenic acid	0.42 ^b^	0.94 ^a^	0.67 ^ab^	0.237	0.012	0.001	0.018
Linoleic acid	12.64 ^c^	35.85 ^a^	23.35 ^b^	0.801	<0.001	<0.001	0.118
Linolenic acid	60.43 ^a^	24.06 ^b^	37.99 ^b^	1.78	<0.001	<0.001	0.055
Arachidic acid	0.09 ^c^	0.40 ^a^	0.29 ^b^	0.045	<0.001	<0.001	0.018
Behenic acid	0.11 ^a^	0.04 ^b^	0.10 ^a^	0.029	0.003	0.016	0.079
Σ Saturated fatty acids	24.81 ^b^	34.28 ^a^	35.13 ^a^	1.934	<0.001	<0.001	0.364
Σ Unsaturated fatty acids	75.19 ^a^	65.73 ^b^	64.87 ^b^	1.934	<0.001	<0.001	0.364

IR: Italian ryegrass monoculture; FB: faba bean monoculture; FBIR: faba bean–Italian ryegrass intercrop (60:40); rsd: residual standard deviation; M: management (crop and fertilization); Y: year^. a,b,c^ Different letters indicate significant differences between treatments according to Duncan’s mean comparison test (*p* < 0.05). Results correspond to mean two agronomic years carried out (2014–2015 y 2015–2016). Saturated acids: caproic; heptanoic; caprylic; capric; lauric; myristic; pentadecanoic; palmitic; heptadecanoic; stearic; arachidic, and behenic acids. Unsaturated acids: palmitoleic; oleic; vaccenic; linoleic; and linolenic.

**Table 3:** Meadow characteristics used in grazing dairy cows in each period of the feeding trials carried out.

	Trial 1 (Year 2015)			Trial 2 (Year 2016)		
	P1	P2	P3	P1	P2	P3
Offer						
Production (kg DM ha^−1^)	2357	1701	1170	1635	895	932
Grass height (cm)	31	22	31	24	25	32
Rejection						
Production (kg DM ha^−1^)	2024	1335	817	1406	502	900
Grass height (cm)	20	17	16	17	18	15
Botanical composition (% DM)						
*Lolium perenne* L.	68.07	9.39	59.50	10.62	33.29	NA
*Trifolium repens* L.	3.86	8.84	-	3.05	3.81	NA
*Trifolium pratensis* L.	-	-	2.78	-	-	NA
*Poa* spp.	1.29	-	-	-	0.57	NA
*Bromus* spp.	-	1.42	-	17.86	11.02	NA
*Agrostis* spp.	7.12	63.16	9.60	-	19.84	NA
*Dactylis glomerata L.*	4.98	-	5.22	0.96	0.48	NA
*Holcus lanatus L.*	-	-	-	2.30	1.24	NA
*Paspalum* spp.	-	-	-	27.10	-	NA
*Cyperus rotundus L.*	-	-	-	0.54	-	NA
*Stellaria media L.*	-	-	-	-	0.05	NA
*Rumex* spp.	0.73	-	-	3.08	-	NA
*Taraxacum* spp.	-	2.64	-	0.66	9.68	NA
*Geranium molle L.*	0.94	0.34	1.78	-	1.62	NA
*Ranunculus* spp.	-	-	-	0.60	-	NA
*Malva* spp.	-	-	-	4.34	-	NA
Other grasses	1.37	3.90	3.09	16.75	6.15	NA
Other dicotyledonous	-	-	2.35	-	-	NA
Unidentifiable remains	-	2.68	5.30	0.60	7.01	NA
Dead matter	11.64	7.63	10.38	11.54	5.24	NA

DM: dry matter; P1: period 1; P2: period 2; P3: period 3; NA: not analysed.

**Table 4:** Nutritional value and energy content of total mixed rations (TMR), extra concentrate (EC), and grass in each trial.

	Trial 1 (Year 2015)							Trial 2 (Yeas 2016)						
	TMR				Grass			TMR				Grass		
	IR	FB	FBIR	EC	P1	P2	P3	IR	FB	FBIR	EC	P1	P2	P3
DM	280.1	370.5	282.0	883.6	147.0	150.9	104.3	280.1	370.5	282.0	883.6	147.0	150.9	104.3
OM	879.0	853.4	882.6	924.2	896.9	898.1	881.5	879.0	853.4	882.6	924.2	896.9	898.1	881.5
CP	139.7	131.5	132.5	200.7	179.1	206.7	221.7	139.7	131.5	132.5	200.7	179.1	206.7	221.7
NDF	483.4	522.5	519.1	215.0	577.3	501.3	540.5	483.4	522.5	519.1	215.0	577.3	501.3	540.5
ADF	334.4	353.4	302.8	95.8	302.1	259.1	266.0	334.4	353.4	302.8	95.8	302.1	259.1	266.0
EE	40.2	24.2	27.8	52.5	NA	NA	NA	40.2	24.2	27.8	52.5	NA	NA	NA
STR	84.2	87.9	101.7	371.7	NA	NA	NA	84.2	87.9	101.7	371.7	NA	NA	NA
NEI	1.39	1.39	1.39	1.93	1.47	1.55	1.45	1.39	1.39	1.39	1.93	1.47	1.55	1.45

IR: TMR made with Italian ryegrass; FB: TMR made with faba bean silage; FBIR: TMR made with faba bean–ryegrass intercrop (60:40); EC: Extra concentrate provided during milking; P1: period 1; P2: period 2; P3: period 3; DM: dry matter (g kg^−1^); OM: organic matter (g kg^−1^ DM); CP: crude protein (g kg^−1^ DM); NDF: neutral detergent fibre (g kg^−1^ DM); ADF: acid detergent fibre (g kg^−1^ DM); EE: ether extract (g kg^−1^ DM); SRT: starch (g kg^−1^ DM); NEI: net energy of lactation (Mcal kg^−1^ DM); NA: not analysed. Results correspond to the average of two feeding trials carried out in 2015 and 2016 except in grass that, due to its variability, is presented by periods.

**Table 5:** Fatty acid profile (g 100 g^−1^ FA) of total mixed rations (TMR) with Italian ryegrass, faba bean, and Italian ryegrass–faba bean intercropped silages.

	IR	FB	FBIR	rsd	*p* (D)	*p* (T)	*p* (D × T)
Caproic acid	0.15 ^b^	0.50 ^b^	2.47 ^a^	1.407	0.030	0.265	0.031
Caprylic acid	0.06	0.13	0.19	0.094	0.101	0.007	0.188
Capric acid	0.02 ^b^	0.04 ^a^	0.04 ^a^	0.006	< 0.001	0.582	0.732
Lauric acid	2.32	1.00	1.10	1.025	0.083	< 0.001	0.077
Myristic acid	0.69 ^b^	0.98 ^a^	0.89 ^a^	0.087	< 0.001	0.562	0.005
Pentadecanoic acid	0.08 ^c^	0.16 ^a^	0.13 ^b^	0.022	< 0.001	< 0.001	0.023
Palmitic acid	26.89	33.26	31.68	4.676	0.086	0.010	0.504
Palmitoleic acid	0.17 ^b^	0.37 ^a^	0.30 ^a^	0.059	< 0.001	< 0.001	< 0.001
Heptadecanoic acid	0.08 ^c^	0.16 ^a^	0.11 ^b^	0.017	< 0.001	0.019	0.003
Stearic acid	1.97 ^b^	2.89 ^a^	2.41 ^ab^	0.415	0.008	< 0.001	0.305
Oleic acid	12.44	16.42	15.65	3.803	0.199	0.490	0.413
Vaccenic acid	0.52 ^b^	0.81 ^a^	0.71 ^ab^	0.165	0.030	< 0.001	0.015
Linoleic acid	26.69	34.93	32.38	5.765	0.077	0.286	0.704
Linolenic acid	27.85 ^a^	8.23 ^b^	11.80 ^b^	4.575	< 0.001	0.783	0.017
Arachidic acid	0.07	0.12	0.08	0.041	0.147	< 0.001	0.315

IR: TMR made with Italian ryegrass; FB: TMR made with faba bean silage; FBIR: TMR made with faba bean–ryegrass intercrop (60:40); rsd: residual standard deviation; D: diet; T: trial. ^a,b,c^ Different letters indicate significant differences between treatments according to Duncan’s mean comparison test (*p* < 0.05). Results correspond to mean two feeding trials carried out (year 2015 and year 2016).

**Table 6:** Initial and final live weight, live weight gains, and food intakes of dairy cows. Milk yields and composition of raw milk from dairy cows according to the type of total mixed ration (TMR) offered.

	IR	FB	FBIR	rsd	*p*
Live body weight (kg)					
Initial live weight	644.4	643.1	640.2	12.950	0.634
Final live weight	648.6	645.1	648.4	13.559	0.648
Live weight gain (kg day^−1^)	0.60	0.29	1.17	2.186	0.490
Intakes (kg DM day^−1^ per cow)					
TMR	10.16	9.21	9.52	1.945	0.398
Concentrate	3.41	3.25	3.40	0.379	0.323
Grass	10.79	11.98	13.33	5.530	0.423
Total	24.57	24.64	26.45	5.510	0.500
Milk yields (kg day^−1^)					
Without standardizing	32.99 ^a^	31.01 ^b^	31.54 ^b^	1.415	<0.001
Standardized for fat and protein	30.46 ^a^	29.41 ^b^	29.19 ^b^	1.216	0.007
Milk composition (g kg^−1^)					
Fat	35.5	37.4	36.0	2.26	0.057
Protein	29.9	30.7	29.8	1.66	0.260
Lactose	47.9	47.4	47.5	1.80	0.679
Solids-non-fat	85.0	84.8	84.4	2.79	0.765
Cells (× 1000 mL^−1^)	63.81	66.44	55.46	34.70	0.607
Urea (mg L^−1^)	225 ^c^	309 ^a^	260 ^b^	30.98	<0.001

IR: TMR made with Italian ryegrass; FB: TMR made with faba bean silage; FBIR: TMR made with faba bean–ryegrass intercrop (60:40); rsd: residual standard deviation. ^a,b,c^ Different letters indicate significant differences between treatments according to Bonferroni test (*p* < 0.05). Results correspond to mean two feeding trials carried out (year 2015 and year 2016).

**Table 7:** Fatty acid profile (g 100 g^−1^ FA) of milk according to the type of total mixed ration (TMR) offered to dairy cows.

Fatty Acid Profile	IR	FB	FBIR	rsd	*p*
Caproic acid	2.35	2.34	2.41	0.253	0.429
Heptanoic acid	0.04 ^a^	0.03 ^b^	0.03 ^b^	0.013	<0.001
Caprylic acid	1.03	0.97	1.01	0.117	0.155
Nonanoic acid	0.04 ^a^	0.02 ^b^	0.02 ^b^	0.013	<0.001
Capric acid	4.00 ^a^	3.61 ^b^	3.96 ^ab^	0.452	0.001
Undecanoic acid	0.11 ^a^	0.06 ^b^	0.07 ^b^	0.041	<0.001
Lauric acid	4.74 ^a^	4.16 ^b^	4.55 ^ab^	0.550	<0.001
Dodecenoic acid	0.09	0.08	0.09	0.031	0.596
Tridecanoic acid	0.14 ^a^	0.11 ^b^	0.11 ^b^	0.033	<0.001
Total short-string	12.53 ^a^	11.39 ^b^	12.24 ^ab^	1.251	0.001
Myristic acid	14.51 ^a^	13.55 ^b^	14.15 ^ab^	1.419	0.020
Myristoleic acid	0.92	0.88	0.83	0.158	0.070
Pentadecanoic acid	1.64 ^a^	1.49 ^ab^	1.43 ^b^	0.198	<0.001
Palmitic acid	40.35 ^a^	37.20 ^b^	39.89 ^a^	3.321	<0.001
Palmitoelaidic acid	0.06 ^b^	0.09 ^a^	0.05 ^b^	0.038	<0.001
Palmitoleic acid	0.84	0.86	0.80	0.126	0.137
Heptadecanoic acid	0.60 ^b^	0.71 ^a^	0.61 ^b^	0.136	0.001
Heptadecenoic acid	0.06 ^a^	0.10 ^a^	0.08 ^ab^	0.034	<0.001
Total middle-string	58.97 ^a^	54.89 ^b^	57.84 ^a^	3.920	<0.001
Stearic acid	10.37 ^b^	12.12 ^a^	11.17 ^ab^	2.039	<0.001
Elaidic acid	0.06	0.05	0.02	0.097	0.293
Trans-Vaccenic acid	1.22 ^b^	2.00 ^a^	1.32 ^b^	0.579	<0.001
Oleic acid	12.24 ^b^	14.08 ^a^	12.82 ^ab^	2.168	0.003
Vaccenic acid	0.38 ^ab^	0.45 ^a^	0.31 ^b^	0.142	<0.001
Linoleic acid	1.87	1.96	1.83	0.349	0.273
Rumenic acid (CLA)	1.25 ^b^	1.80 ^a^	1.26 ^b^	0.538	<0.001
Linolenic acid	0.98	1.08	1.04	0.234	0.237
Arachidic acid	0.04 ^b^	0.06 ^a^	0.05 ^b^	0.039	0.044
Behenic acid	< LC	0.01	< LC	0.006	0.121
Arachidonic acid	0.03	0.04	0.03	0.022	0.058
Eicosapentaenoic acid	0.01	0.01	0.01	0.010	0.085
Docosapentaenoic acid	0.01	0.01	0.01	0.013	0.177
Total long-string	28.46 ^b^	33.67 ^a^	29.88 ^b^	4.679	<0.001

IR: TMR made with Italian ryegrass; FB: TMR made with faba bean silage; FBIR: TMR made with faba bean–ryegrass intercrop (60:40); rsd: residual standard deviation; <LC below quantification level. ^a,b^ Different letters indicate significant differences between treatments according to Bonferroni test (*p* < 0.05). Results correspond to mean two feeding trials carried out (year 2015 and year 2016).

**Table 8:** Sum of fatty acids (g 100 g^−1^ FA) according to the number of double bonds and fatty acid ratios.

Fatty Acid Profile	IR	FB	FBIR	rsd	*p*
Σ Saturated acids	79.95 ^a^	76.45 ^b^	79.46 ^a^	2.906	<0.001
Σ Unsaturated acids	20.02 ^b^	23.50 ^a^	20.50 ^b^	2.894	<0.001
Σ Monounsaturated acids	15.87 ^b^	18.59 ^a^	16.32 ^b^	2.435	<0.001
Σ Polyunsaturated acids	4.15 ^b^	4.91 ^a^	4.19 ^b^	0.906	<0.001
Saturated/Unsaturated ratio	4.08 ^a^	3.37 ^b^	4.13 ^a^	0.772	<0.001
Omega 6 (ω-6)	1.90	2.00	1.86	0.354	0.212
Omega 3 (ω-3)	1.00	1.10	1.06	0.237	0.238
ω-6/ω-3 ratio	2.20	1.93	1.97	0.603	0.215
Trans-vaccenic/Vaccenic ratio	2.71 ^b^	4.39 ^a^	3.59 ^ab^	1.735	<0.001

IR: TMR made with Italian ryegrass; FB: TMR made with faba bean silage; FBIR: TMR made with faba bean–ryegrass intercrop (60:40); rsd: residual standard deviation; <LC below quantification level. ^a,b^ Different letters indicate significant differences between treatments according to Bonferroni test (*p* < 0.05). Results correspond to mean two feeding trials carried out (year 2015 and year 2016). Saturated acids: caproic, heptanoic, caprylic, nonanoic, capric, undecanoic, lauric, tridecanoic, myristic, pentadecanoic, palmitic, heptadecanoic, stearic, arachidic, and behenic acids. Unsaturated acids: dodecenoic, myristoleic, palmitoelaidic, palmitoleic, heptadecenoic, elaidic, trans-vaccenic, oleic, vaccenic, linoleic, rumenic, linolenic, arachidonic, eicosapentaenoic, and docosapentaenoic acids. Monounsaturated acids: dodecenoic, myristoleic, palmitoelaidic, palmitoleic, heptadecenoic, elaidic, trans-vaccenic, oleic, and vaccenic acids. Polyunsaturated acids: linoleic, rumenic, linolenic, arachidonic, eicosapentaenoic, and docosapentaenoic acids. Omega 3 acids: linolenic, eicosapentaenoic, and docosapentaenoic acids. Omega 6 acids: linoleic and arachidonic acids.

## Data Availability

All data used to support the findings of this study are included within the article.
